# Macrolide Resistance in *Streptococcus pneumoniae*

**DOI:** 10.3389/fcimb.2016.00098

**Published:** 2016-09-21

**Authors:** Max R. Schroeder, David S. Stephens

**Affiliations:** ^1^Departments of Medicine, Emory UniversityAtlanta, GA, USA; ^2^Departments of Microbiology and Immunology, Emory UniversityAtlanta, GA, USA; ^3^Departments of Epidemiology, Emory UniversityAtlanta, GA, USA

**Keywords:** *Streptococcus pneumoniae*, antibiotic resistance, macrolide resistance, *erm*(B), *mef*(A/E)/*mel*(*msr*(D)), Mega, pneumococci

## Abstract

*Streptococcus pneumoniae* is a common commensal and an opportunistic pathogen. Suspected pneumococcal upper respiratory infections and pneumonia are often treated with macrolide antibiotics. Macrolides are bacteriostatic antibiotics and inhibit protein synthesis by binding to the 50S ribosomal subunit. The widespread use of macrolides is associated with increased macrolide resistance in *S. pneumoniae*, and the treatment of pneumococcal infections with macrolides may be associated with clinical failures. In *S. pneumoniae*, macrolide resistance is due to ribosomal dimethylation by an enzyme encoded by *erm*(B), efflux by a two-component efflux pump encoded by *mef* (E)/*mel*(*msr*(D)) and, less commonly, mutations of the ribosomal target site of macrolides. A wide array of genetic elements have emerged that facilitate macrolide resistance in *S. pneumoniae*; for example *erm*(B) is found on Tn*917*, while the *mef* (E)/*mel* operon is carried on the 5.4- or 5.5-kb Mega element. The macrolide resistance determinants, *erm*(B) and *mef* (E)/*mel*, are also found on large composite Tn*916*-like elements most notably Tn*6002*, Tn*2009*, and Tn*2010*. Introductions of 7-valent and 13-valent pneumococcal conjugate vaccines (PCV-7 and PCV-13) have decreased the incidence of macrolide-resistant invasive pneumococcal disease, but serotype replacement and emergence of macrolide resistance remain an important concern.

## Introduction

*Streptococcus pneumoniae*, the pneumococcus, is a commensal of the human nasopharynx and an opportunistic pathogen that is a leading worldwide cause of death for children under the age of 5 years (Walker et al., 2013). In addition to localized infections such as otitis media and pneumonia, the pneumococcus may cause severe invasive disease (IPD) including bacteremia and meningitis. Development of penicillin resistance in the pneumococcus in the 1980s–1990s shifted antibiotic treatment of suspected pneumococcal upper respiratory infections and pneumonia to macrolides. Widespread macrolide use, however, is associated with increased macrolide resistance in *S. pneumoniae* (Bergman et al., 2006; Malhotra-Kumar et al., 2007). Clinical failures of macrolide treatment of pneumococcal infections have been reported for lower respiratory tract infections (Klugman, 2002) and bacteremia (Lonks et al., 2002; Schentag et al., 2007). Widespread macrolide use is a strong selective pressure contributing to the expansion of macrolide-resistant *S. pneumoniae* (Bergman et al., 2006; Keenan et al., 2015). Globally, macrolide resistance among *S. pneumoniae* is geographically variable but ranges from <10% to >90% of isolates (Farrell et al., 2008; Pan et al., 2015; Xiao et al., 2015).

## Macrolide antibiotics

Macrolides are defined by a complex macrocyclic structure with a 14-, 15-, or 16-membered lactone ring substituted with neutral or amino sugar groups. Macrolides inhibit bacterial protein synthesis by binding to the large 50S ribosomal subunit and disrupting protein elongation by causing the dissociation of the peptidyl-tRNA.

Erythromycin, discovered in 1952, is a 14-membered macrolide produced by *Saccharopolyspora erythraeus* (formerly *Streptomyces erythraeus*; McGuire et al., 1952). After the discovery of erythromycin and other naturally-produced macrolides, research focused on the creation of synthetic and semisynthetic macrolides (Kirst, 2010; Seiple et al., 2016). Azithromycin and clarithromycin are semisynthetic macrolides approved for use in the United States, and azithromycin is one of the most prescribed antibiotics in the US (Hicks et al., 2015). Additional macrolides such as roxithromycin and josamycin are approved in other countries worldwide. Macrolides bind reversibly to the 23S rRNA at a site near the peptidyl transferase center of the 50S ribosomal subunit (Kannan and Mankin, 2011). Macrolide binding occurs in pre-structured ribosomal assemblies (Pokkunuri and Champney, 2007). The smaller macrolides (14- and 15-membered) partially block the nascent peptide channel to inhibit the elongating peptide chain while larger macrolides (16-membered) fully block the nascent peptide channel and cause ribosomal disassociation that reversibly inhibits protein synthesis (Weisblum, 1995b). Though distinct in chemical structure, the lincosamide and streptogramin class antibiotics have overlapping binding sites with macrolides and have similar mechanisms of action (Kirst, 2010).

## Mechanisms of macrolide resistance

### Ribosomal modification

Erythromycin ribosomal methylase (*erm*) family genes encode adenine-specific N-methyltransferases that methylate the 23S rRNA to prevent antibiotic binding (Weisblum, 1995a). The ribosomal methylase found in *S. pneumoniae* is primarily encoded by *erm*(B) whose gene product dimethylates the target site of the 23S rRNA (A2058 in *Escherichia coli*; Skinner et al., 1983; Johnston et al., 1998). The *erm*(B) gene is the most common macrolide resistance determinant in *S. pneumoniae* (Table 1). Erm(A) subclasses *erm*(A) (Syrogiannopoulos et al., 2001) and *erm*(TR) (Camilli et al., 2008) are rarely found in *S. pneumoniae*. Ribosomal methylation by Erm(B) confers resistance to macrolides, lincosamides, and streptogramin B, which is characterized as the MLS_B_ phenotype (Weisblum, 1995a). In addition to the expanded spectrum of resistance, *erm*(B) provides high-level resistance to macrolides (erythromycin MICs usually ≥256 μg/ml).

**Table 1:** **Distribution of macrolide resistance genotypes by country**.

**Continent Country**	**Macrolide resistance genotype distribution %**						**Years**	**References**
	***erm*(B)**	***mef*(A/E)**	***mef*(E)**	***mef*(A)**	***erm*(B) + *mef*(E)**	**Negative^a^**		
**AFRICA**								
Morocco	90.2		6.5	0	3.3	0	2007–2014	Diawara et al., 2016
South Africa	36.5	16.3			46.4	0.4	2003–2004	Farrell et al., 2008
**ASIA**								
China	63.4	0.6			36.0	0	2006–2008	Ma et al., 2013
	69.6	0			30.4		2010	Zhou et al., 2011
	62.9				37.1		2012–2013	Geng et al., 2014
Hong Kong	45.9	27.7			26.4	0	2008–2009	Kim et al., 2012
Iran	44	16			40		2011–2013	Azadegan et al., 2015
Japan	56.3	28.8			10.9	4.0	2011	Kawaguchiya et al., 2014
	59.5	25.2			12.6	2.7	2011–2012	Okade et al., 2014
	53.2	21.8			17.9	7.1	2012	Chiba et al., 2014
Jordan	26.4	24.5			13.2	35.8	2012–2013	Swedan et al., 2016
Lebanon	65.3	0			19.5	14.6	2005–2009	Daoud et al., 2011
	36.4	18.1			31.8	13.6	2008–2010	Taha et al., 2012
Malaysia	35.2	42.6			9.3	13.0	2008–2009	Kim et al., 2012
Saudi Arabia	37.5	62.5			0	0	2003–2004	Farrell et al., 2008
Sri Lanka	73.3	13.3			13.3	0	2008–2009	Kim et al., 2012
South Korea	43.3	13.0			43.3	0.4	2008–2009	Kim et al., 2012
Taiwan	55.1	22.5			21.4	1.0	2008–2009	Kim et al., 2012
	70.0	5.0			25.0		2010	Safari et al., 2014
Thailand	47.9	37.2			11.7	3.2	2008–2009	Kim et al., 2012
Vietnam	56.9	2.1			41.0	0	2008–2009	Kim et al., 2012
**AUSTRALIA**								
Australia	32.4		3.9	20.6	35.3 (6.9)^b^	0.9	2005	Xu et al., 2010
**EUROPE**								
Austria	45.5	54.5			0	0	2003–2004	Farrell et al., 2008
Belgium	90.2	1.6			3.3	4.9	2007–2009	Lismond et al., 2012
Bulgaria	63.2	21.0			15.8		2006–2010	Setchanova et al., 2012
Denmark	30.4		15.9	49.3 (1.4)^c^	0	2	2007	Nielsen et al., 2010
France	90.0	2.4			1.2	6.5	2003–2004	Farrell et al., 2008
Finland	30.5		40.4	15.7	0.9	12.6	2002–2006	Siira et al., 2009
Germany	27.0		11.2	57.7	4.1	0	2005–2006	Bley et al., 2011
	66.8		8.3	20.7	3.6		2012–2013	Imöhl et al., 2015
Greece	22.0		45.8	0	32.2		2009	Grivea et al., 2012
Hungary	82.4	11.8			5.9	0	2003–2004	Farrell et al., 2008
Ireland	38.9	61.1			0	0	2003–2004	Farrell et al., 2008
Italy	55.8	38.5			1.0	4.8	2003–2004	Farrell et al., 2008
Poland	80.8	7.7			3.8	7.7	2003–2004	Farrell et al., 2008
Russia	54.1	12.7			30.6	2.6	2009–2013	Mayanskiy et al., 2014
Slovak Republic	64.7	5.9			17.6	11.8	2003–2004	Farrell et al., 2008
Spain	74.3	7.7			17.9		2000–2007	De La Pedrosa et al., 2009
Switzerland	70.6	23.5			0	5.9	2003–2004	Farrell et al., 2008
Turkey	44.4	11.1			44.4		2008–2009	Sirekbasan et al., 2015
United Kingdom	20.8	70.8			4.2	4.2	2003–2004	Farrell et al., 2008
**NORTH AMERICA**								
Canada	27.0	50.0	95	5	19.0	3.6	1997–2002	Wierzbowski et al., 2005b
							2008	Wierzbowski et al., 2014
Mexico	17.2	72.4			10.3	0	2003–2004	Farrell et al., 2008
USA	19.5	51.3			28.7	0.5	2007	Hawkins et al., 2015
Alaska	15.0	58.8			20.0	6.3	2006–2010	Rudolph et al., 2013
Arizona	5		25	2.5	67.5		2007–2008	Bowers et al., 2012
**SOUTH AMERICA**								
Argentina	19.2	76.9			3.8		2009–2010	Reijtman et al., 2013
Brazil	36.0	44.0			20.0		2007–2012	Caierão et al., 2014
Colombia	56.9	40.2	30.7	0.9	7.1	4.4	1994–2008	Ramos et al., 2014
	53.7				0	6.1	2005–2008	Hidalgo et al., 2011
Peru	53.3	33.3			0	13.3	2003–2004	Farrell et al., 2008
Venezuela	83.3		12.5	0.0	4.2		2007	Quintero et al., 2011

a *PCR negative for erm(B), mef(A/E), mef(A), and mef(E). Some authors have determined these to be ribosomal mutations*.

b *Strains contain both erm(B) and mef(A)*.

c *Strains contain mef(I)*.

The induction of *erm*(B) allows high-level translation of Erm(B) in the presence of inducers such as erythromycin (Chancey et al., 2011). In the pneumococcus, *erm*(B) expression may be inducible or constitutively expressed to high levels. As expression of *erm* genes is repressed in the absence of inducing drugs through a mechanism of translational attenuation; *erm*(B) expression has been proposed to have a bacterial fitness cost (Min et al., 2008; Chancey et al., 2012; Gupta et al., 2013). A recent study found that a *Staphylococcus aureus* strain expressing *erm*(C) was outcompeted by *S. aureus* expressing catalytically-inactive *erm*(C) (Gupta et al., 2013), supporting the need for tight regulation of expression. Interestingly, deletion of the leader sequence of *erm*(B) in *S. pneumoniae* was found to confer resistance to telithromycin, the first-generation ketolide, a semi-synthetic macrolide antibiotic, by allowing constitutive expression (Wolter et al., 2008).

### Macrolide efflux

Macrolide efflux in *S. pneumoniae* has been the most common cause of macrolide resistance in North America, the United Kingdom, and others (Table 1). Pneumococcal macrolide efflux is encoded by the *mef* (E)/*mel* operon and occurs through an as yet incompletely understood mechanism of macrolide binding and membrane targeting for efflux (Chancey et al., 2012). Macrolide resistance in *S. pneumoniae* requires both *mef* (E) and *mel*. These genes are carried on the macrolide efflux genetic assembly (Mega) element and are expressed from a single promoter inducible by 14- and 15-membered macrolides (e.g., erythromycin, clarithromycin, and azithromycin; Gay and Stephens, 2001; Ambrose et al., 2005; Chancey et al., 2015b). Expression of *mef* (E) and *mel* is tightly controlled through transcriptional attenuation (Chancey et al., 2015b).

The first gene, *mef* (E) shares 90% sequence identity with *mef* (A) from *Streptococcus pyogenes* (Tait-Kamradt et al., 1997; Roberts et al., 1999). While *mef* (E) is most common, *mef* (A) is more common in Germany, Denmark, and Australia (Table 1). Another homolog, *mef* (I), also shares 91% identity with *mef* (A), has been found in *S. pneumoniae* (Cochetti et al., 2005; Wierzbowski et al., 2005b) but is rarely found (Table 1). Most studies do not distinguish between *mef* (E) and *mef* (A) and thus report only *mef* (E) or *mef* (A) rather than *mef* (A/E), which is a more accurate description of the data. In *S. pneumoniae, mef* (E) encodes a 405 amino acid protein that belongs to the major facilitator superfamily, which utilizes proton motive force-driven efflux to expel molecules from cells (Tait-Kamradt et al., 1997). The second gene, *mel* (also known as *msr*(D)) is a homolog of the *S. aureus* gene *mrs*(*A*) (Roberts et al., 1999), which encodes an ATP-binding cassette (ABC) transporter protein but lacks typical hydrophobic, membrane-binding domains, and is predicted to interact with chromosomally encoded transmembrane complexes (Ambrose et al., 2005). Mef(E) and Mel have been shown to synergistically provide macrolide resistance and operate as a two-component efflux pump in *S. pneumoniae* (Ambrose et al., 2005; Zhang et al., 2016). A recent *E. coli* study suggests a physical interaction between Mef(E) and Mel and that Mel may bind macrolides and localize to the membrane (Nunez-Samudio and Chesneau, 2013). In *S. pyogenes* the presence of *msr*(D) alone was required for macrolide resistance (Zhang et al., 2016) and recent evidence suggests antibiotic resistance by ATP-binding cassette proteins may occur through ribosomal protection by displacing ribosome-bound macrolide molecules (Sharkey et al., 2016). Thus, the working model for macrolide efflux in *S. pneumoniae* may be macrolide displacement from ribosomes by *mel*, which transfers macrolide molecules to *mef* (E) for efflux.

*S. pneumoniae* with *mef* (E)/*mel* have been shown to have an M phenotype, which is resistant to 14- and 15-membered macrolides but susceptible to lincosamides and streptogramin B (Tait-Kamradt et al., 1997). While *mef* (E)/*mel*-containing strains display low level resistance (MICs 1–8 μg/ml) to erythromycin, macrolide induction increases expression of *mef* (E)/*mel* and results in increased levels of macrolide resistance (Wierzbowski et al., 2005a). Induction of *mef* (E)/*mel* by macrolides increases MICs to ≥16 μg/ml (Ambrose et al., 2005; Chancey et al., 2011). The presence of the two-component efflux pump encoded by *mef* (E)/*mel* also increases resistance to the human antimicrobial peptide LL-37 (Zähner et al., 2010). LL-37 also induces expression of the efflux pump (Zähner et al., 2010). These data may suggest the efflux pump is induced during nasopharyngeal colonization and primes the *mef* (E)/*mel*-containing pneumococci to resist macrolide antibiotics.

### Ribosomal mutations

Point mutations in 23S rRNA at or near the macrolide binding residue A2058 (*E. coli* ribosome) have resulted in high-level macrolide resistance (Vester and Douthwaite, 2001; Franceschi et al., 2004). Mutations of ribosomal proteins L4 and L22 confer macrolide resistance in pathogenic and nonpathogenic bacteria including pneumococci. L4 and L22 are ribosomal proteins with domains on the surface of the ribosome as well as tentacles that extend into the exit tunnel in proximity to the macrolide-binding site (Schuwirth et al., 2005). In *E. coli*, a Lys-63-Glu change in the L4 protein (*rplD*) as well as a triple amino acid deletion of Met-82, Lys-83, and Glu-84 from L22 (*rplV*) confer resistance to macrolides (Wittmann et al., 1973; Chittum and Champney, 1994). A variety of additional L4 and L22 mutations have also been found to confer macrolide resistance (Zaman et al., 2007; Diner and Hayes, 2009). While the overall incidence is rare in *S. pneumoniae*, L4 and L22 mutations have been shown to result in macrolide resistance (Franceschi et al., 2004).

### Dual macrolide resistance genotype

*S. pneumoniae* containing both *erm*(B) and *mef* (E)/*mel* were first reported in the late-1990s (Corso et al., 1998; Nishijima et al., 1999) and are now found worldwide (Farrell et al., 2008). The dual macrolide resistance genotype occurred in 12% of global isolates collected from 2003 to 2004, which is twice the frequency reported from 1999 to 2000 (Farrell et al., 2008). In 2004, 18.4% of *S. pneumoniae* isolates from the US were found to have the dual *erm*(B) and *mef* (E)/*mel* genotype (Jenkins et al., 2008); in a recent study, up to 52% of macrolide-resistant isolates from Arizona were found to contain both macrolide resistance genes (Bowers et al., 2012). Tn*2010* has been identified as the major composite mobile element that contains *erm*(B) and *mef* (E)/*mel* (Mega) (Del Grosso et al., 2006). Following introduction of the 7-valent pneumococcal conjugate vaccine (PCV-7) the “replacement” serotype 19A (ST320) with Tn*2010* emerged (Del Grosso et al., 2007). ST320 is a multidrug resistant strain that appears to represent a “capsule switch” from serotype 19F and is responsible for a global pandemic in the wake of PCV-7 introduction (Moore et al., 2008; Li et al., 2011). The high-level and broader resistance conferred by *erm*(B) would predict that *mef* (E)/*mel* is functionally redundant in *erm*(B)-containing *S. pneumoniae*.

## Dissemination of resistance determinants

### Macrolide resistance chromosomal locations

The *mef* (E)/*mel*-containing genetic element Mega is found in at least six distinct chromosomal sites within the pneumococcal genome (Chancey et al., 2015a), while *mef* (A) is found on Tn*1207.1* (Xu et al., 2010). Mega insertion sites are distributed around the chromosome: (I) a phosphomethylpyrimidine kinase (TIGR4 SP_1598), (II) a DNA-3-methyladenine glycosylase (SP_0180), (III) a capsule biosynthesis gene (SP_0103), (IV) the RNA methyltransferase *rumA* (SP_1029) (Gay and Stephens, 2001), (V) *orf6* of Tn*916*-like elements (Del Grosso et al., 2006), and (VI) a novel insertion into a *S. suis* homolog element found in *S. pneumoniae* (Chancey et al., 2015a). Due to genetic variations at insertion site IV, this class is subdivided: (IVa) Mega and IS*Smi* element insertion along with deletion of the 30.7 kb pneumococcal pathogenicity island-1 (PPI-1), and (IVb) simple insertion of Mega into *rumA* with PPI-1 intact, and (IVc) same organization as IVa with a *S. equi* subspecies *zooepidemicus*-related integrative and conjugative element (42 kb) inserted upstream of Mega (Chancey et al., 2015a).

The Mega element lacks genes required for transposition (Gay and Stephens, 2001). Analysis of the Mega insertion sites revealed a putative target sequence of 5′-TTTCCNCAA-3′ about six base pairs upstream of the insertion and Tn*916*-like coupling sequences (Chancey et al., 2015a). The genes required for Mega transposition may be present on other conjugative elements of the pneumococcal genome and in non-*S. pneumoniae* commensal organisms (Gay and Stephens, 2001; Chancey et al., 2015a). While Mega is infrequently transferred through transposition, once stabilized in the genome Mega is widely disseminated through horizontal DNA exchange and homologous recombination.

Tn*916* is the prototype conjugative transposon that contains the tetracycline resistance gene *tet*(M), and is found in many Gram-positive bacteria. Tn*916* may incorporate additional antibiotic resistance determinants which comprise larger Tn*916*-like composite elements (Roberts and Mullany, 2011). The history and molecular mechanisms of the Tn*916* family are beyond the scope of this review, but have been explored previously (Roberts and Mullany, 2009). The most common Tn*916*-like elements in *S. pneumoniae* containing erythromycin resistance cassettes include Tn*2009*, Tn*6002*, and Tn*2010* (Chancey et al., 2015a). Tn*2009* is a Tn*916*-like element with Mega inserted into *orf* 6 of Tn*916* to provide macrolide resistance, the M phenotype (Del Grosso et al., 2004). Tn*6002* is also a Tn*916*-like element with macrolide resistance, with a MLS_B_ phenotype due to the incorporation of an *erm*(B)-containing element into *orf20* of Tn*916* (Brenciani et al., 2007). The *erm*(B) gene may also be incorporated into Tn*916*. Tn*917*, an *erm*(B)-containing transposon insertion into *orf9* of Tn*916* creates Tn*3872* (Brenciani et al., 2007). *S. pneumoniae* with the dual macrolide resistance genotype most often contain Tn*2010* or rarely the newly described element Tn*2017* (Del Grosso et al., 2009). Tn*2010* is a Tn*916*-like element with Mega in *orf6* and the *erm*(B) element in *orf20* of Tn*916* (Del Grosso et al., 2007). Tn*2010* likely arose through the homologous recombination of Tn*2009* with Tn*6002* (Chancey et al., 2015a). A similar recombination event likely occurred with Tn*2009* and Tn*387*2 to create Tn*2017*, which is a Tn*916*-like element with a Mega insertion in *orf6* and Tn*917* in *orf9* of Tn*916* (Del Grosso et al., 2009).

### Interspecies exchange of macrolide resistance

During the growth cycle, pneumococci develop a natural state of competence and can acquire DNA from the environment. A mechanism of DNA repair allows for integration of new DNA through homologous recombination (Straume et al., 2015). The human nasopharynx is the primary ecological niche for the pneumococcus during asymptomatic carriage (Simell et al., 2012), where *S. pneumoniae* has the opportunity to acquire DNA from other pneumococci and from commensal bacteria of the upper respiratory tract that may act as a reservoir for antibiotic resistance.

Other bacteria that reside in the human upper respiratory tract carry the macrolide resistance genes. Tn*6002* is the most common *erm*(B)-containing mobile genetic element of *S. pyogenes* (Brenciani et al., 2007). A recent study found Mega, Tn*2009*, Tn*6002*, and Tn*2010* in commensal viridans group streptococci isolated from the human oropharynx (Brenciani et al., 2014). In this study, *S. mitis* was the most commonly isolated streptococcal species with the macrolide resistance elements. Other Gram-positive bacteria have been shown to carry *erm*(B) and/or *mef* (E) (Luna et al., 1999; Seppälä et al., 2003; Santoro et al., 2014). The Tn*2009* element has been found in commensal, Gram-negative *Acinetobacter junii*, and there is evidence of this Mega-containing transposon in other Gram-negative species including *E. coli, Enterobacter cloacae, Klebsiella* sp., *Proteus* sp., and *Pseudomonas* sp. (Ojo et al., 2006). Interspecies dissemination of mobile genetic elements containing antibiotic resistance cassettes appears common.

Asymptomatic pneumococcal carriage occurs in children and adults with rates in children ranging from <15% to >90% in developing countries (Shak et al., 2013). Carriage varies based on factors including geography and socioeconomic class (O'Brien and Nohynek, 2003; Simell et al., 2012). During nasopharyngeal carriage, *S. pneumoniae* forms biofilms that enhance natural transformation (Chao et al., 2014) and genetic exchange during co-colonization by two pneumococcal strains is efficient with transformation efficiencies up to 10^−2^ (Marks et al., 2012). This environment may have allowed for the dissemination of macrolide resistance determinants including the assembly and selection of the dual macrolide resistance elements, e.g., Tn*2017* and the more common Tn*2010* (discussed above).

## Impact of pneumococcal conjugate vaccines on macrolide resistance

Between 1994 and 1999, macrolide-resistant invasive pneumococcal disease (MR-IPD) rapidly emerged in the US largely due infections caused by isolates containing *mef* (E)/*mel* (Gay et al., 2000; Stephens et al., 2005). Introduction of PCV-7 in 2000 significantly reduced the incidence of MR-IPD in the US as the highest rates of macrolide resistance were present in PCV-7 vaccine serotypes (Stephens et al., 2005; Rudolph et al., 2013; Hawkins et al., 2015). Similar vaccine specific reductions were observed worldwide, which was observed in Germany through the reduction of *mef* (A)-containing serotype 14 (ST9) isolates (Bley et al., 2011; Imöhl et al., 2015). The incidence of MR-IPD from 2002 through 2009 stabilized while macrolide-resistant PCV-7 serotypes continued to decline; this decline was offset by the rapid emergence of macrolide-resistant serotypes not covered by PCV-7, specifically serotype 19A, ST320 (formerly CC271; Del Grosso et al., 2007; Bowers et al., 2012; Chancey et al., 2015a).

The incidence of MR-IPD caused by serotype 19A isolates with the dual macrolide resistance phenotype (both *erm*(B) and *mef* (E)/*mel*) rapidly increased from 2003 through 2010 in the US and worldwide (Li et al., 2011; Quintero et al., 2011; Bowers et al., 2012; Sharma et al., 2013; Pan et al., 2015; Lyu et al., 2016). Selective pressure by PCV-7 and the continued high-level use of macrolides provided an opportunity for this 19A clone to expand worldwide. The introduction of PCV-13 in the later-2010, which contains serotype 19A, was successful in reducing carriage and IPD caused by vaccine serotypes including macrolide-resistant serotype 19A isolates (Desai et al., 2015; Imöhl et al., 2015). Overall, pneumococcal conjugate vaccination has yielded sustained reductions in pneumococcal disease (Pilishvili et al., 2010). Despite challenges with serotype replacement, PCVs are an effective intervention in reducing the incidence of disease caused by macrolide-resistant pneumococcal serotypes contained in the vaccine. Continued expansion of pediatric pneumococcal vaccination into developing countries is predicted to greatly reduce the global burden of pneumococcal disease and antibiotic resistant pneumococci (Rodgers and Klugman, 2011).

## Conclusions

Macrolide resistance rapidly emerged in *S. pneumoniae* in the early-1990s. The introduction and widespread use of semisynthetic macrolides including azithromycin and clarithromycin were important drivers of macrolide resistance in pneumococci. Macrolide resistance in *S. pneumoniae* is predominantly due to ribosomal methylation by the gene product encoded by *erm*(B) and macrolide efflux by a two-component efflux pump encoded by *mef* (E)/*mel* on the transformable genetic element Mega. Both of these macrolide resistance determinants are associated with larger composite elements (i.e., Tn*6002* and Tn*2009*) and can be found on the same element. PCVs are effective in reducing macrolide resistance caused by vaccine serotypes and thus have been effective in the reduction of MR-IPD caused by vaccine strains. But “serotype replacement” has been an issue (e.g., 19A) and emergence of macrolide resistance in new serotypes is a concern. Continued research is needed to better understand the mechanism of macrolide efflux by Mef(E)/Mel, the emergence of genetic elements containing both *erm*(B) and *mef* (E)/*mel*, and to continue surveillance to monitor new changes in macrolide resistance in pneumococci.

## Author contributions

MS wrote the paper and MS and DS developed and edited the paper.

## Funding

The work was supported by Emory University (Ph. D. thesis).

### Conflict of interest statement

The authors declare that the research was conducted in the absence of any commercial or financial relationships that could be construed as a potential conflict of interest.
